# *Culicoides* biting midges (Diptera: Ceratopogonidae) feeding on donkeys in the United Kingdom, with reference to the risk of transmission and persistence of African horse sickness virus

**DOI:** 10.1111/mve.70061

**Published:** 2026-03-20

**Authors:** Zoe Langlands, Simon Gubbins, Simon Carpenter, Marion England

**Affiliations:** ^1^ The Pirbright Institute Surrey UK

**Keywords:** African horse sickness virus, Ceratopogonidae, *Culicoides*, donkey, Orbivirus

## Abstract

African horse sickness virus (AHSV: Sedoreoviridae; Orbivirus) causes a severe and often fatal disease in horses (African horse sickness: AHS) and is transmitted almost exclusively by *Culicoides* biting midges (Diptera: Ceratopogonidae). In recent years, unprecedented outbreaks of AHSV have occurred in new geographical foci in Thailand and other related *Culicoides*‐borne viruses continue to emerge unexpectedly, causing disease outbreaks in northern Europe. This study investigated *Culicoides* abundance and diversity at a donkey (*Equus asinus*) sanctuary in southern England. The incidence and severity of AHS in infected donkeys are lower than in horses, with concerns, therefore, that these species could act as potential reservoirs in the event of an incursion of AHSV. A total of 21,350 *Culicoides* of 20 species were collected over 14 nights during spring and summer 2019 using three Onderstepoort Veterinary Institute ultraviolet light‐suction traps. The most abundant species were identified within the subgenus *Avaritia* (19,574; 91.7%), which are known vectors of other Orbiviruses in northern Europe and have been previously identified as putative vectors of AHSV in southern Europe. Furthermore, *Culicoides* blood‐feeding on donkeys was confirmed for the subgenus *Avaritia* through polymerase chain reaction of blood‐fed female *Culicoides* using a 685 bp region of the cytochrome *c* oxidase subunit 1 gene. Data on the size and distribution of the donkey population and the potential impact of infection with AHSV on donkeys within the United Kingdom are scarce. This study demonstrates that large populations of *Culicoides* can exist near these hosts and that they regularly take blood meals from them. There is a potential risk that donkeys could play a significant role in transmission and persistence of AHSV in the event of an incursion into the United Kingdom, which could complicate disease control.

## Graphical abstract

- *Culicoides* biting midges were collected at a donkey sanctuary in the United Kingdom using UV light‐suction traps.
- *Culicoides* were found in abundance and all specimens were identified to species level. Blood‐feeding on donkeys was confirmed by polymerase chain reaction (PCR) and sequencing of host bloodmeals.
- Donkeys could play a significant role in the transmission and persistence of African horse sickness virus in the event of an incursion into the United Kingdom.

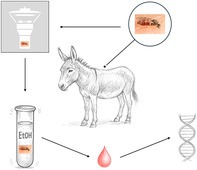

## INTRODUCTION

*Culicoides* biting midges (Diptera: Ceratopogonidae) are biological vectors of arthropod‐borne viruses (arboviruses) that can cause diseases of global veterinary importance in livestock and equids (Purse et al., 2015). Certain *Culicoides* species can transmit African horse sickness virus (AHSV) which causes the disease African horse sickness (AHS) in equids. There are nine known serotypes of AHSV (Howell, 1962) and clinical disease can manifest from a mild fever to severe and often fatal pulmonary disease (WOAH, 2020b). Mild fever typically occurs in the primary maintenance reservoirs of AHSV, which are the three extant species of zebra and the African wild ass (*Equus africanus*), or in previously infected and recovered horses (Barnard, 1993; Theiler, 1921). The more serious forms of AHS typically occur in naïve horses and domestic donkeys (*Equus asinus*) (Ayelet et al., 2013; Scacchia et al., 2015) with case fatality among naïve horse populations as high as 90% (Mellor & Hamblin, 2004).

AHS is endemic in sub‐Saharan Africa (Dennis et al., 2019). Few outbreaks have been recorded outside of Africa, with the most recent being in Thailand in March 2020 (King et al., 2020), most likely as a result of the importation of AHS‐infected animals (Bunpapong et al., 2021; Castillo‐Olivares, 2021). Peninsular Malaysia also reported five cases in backyard horses in September 2020 (WOAH, 2020a). Within Europe, there have been two outbreaks of AHS: The first in 1966–7 in southern Spain caused by AHSV‐9, and the second caused by AHSV‐4 in 1987–90 in Spain and Portugal, following the importation of AHSV‐infected zebra (Carpenter et al., 2017).

Within endemic regions, the primary vector of AHSV is *Culicoides imicola* Kieffer, and to a lesser extent, *Culicoides bolitinos* Meiswinkel (Meiswinkel et al., 2000). Additionally, two northern European species, *Culicoides obsoletus* Meigen and *Culicoides pulicaris* Linnaeus, known to be present in the United Kingdom, were implicated as possible vectors of AHSV during outbreaks in Spain (Mellor et al., 1990). These species, which are abundant at stables across northern Europe, are considered to be a risk factor for AHSV transmission (Mellor & Boorman, 1995; Robin et al., 2014; Carpenter et al., 2017). Further, vector competence for AHSV‐4 and AHSV‐9 of field‐caught *C. obsoletus* and *Culicoides scoticus* Downes and Kettle from Switzerland has been demonstrated (Maurer et al., 2021).

AHSV is a major threat to equine sport and companion animal industries (Carpenter et al., 2017) with the horse population in the United Kingdom currently estimated to be 850,000 animals (British Equestrian, 2023). The UK horse industry is valued at £5 billion and employs around 230,000 people (British Equestrian, 2023). Available data on the distribution and abundance of other equine species in the United Kingdom are limited, including those of populations of domestic donkeys and zebra.

According to a survey of donkey carers in the United Kingdom, there were an estimated 8954 donkeys registered in 2007, but this is considered an underestimate (Cox et al., 2010). Indeed, other anecdotal evidence puts the total UK donkey population at around 27,000 (Brown, 2023). Donkeys are not considered to be long‐term reservoir hosts for AHSV as viraemia in this species has only been observed to last for up to 12 days post infection (Hamblin et al., 1998). However, the potential role of donkeys during an outbreak in Northern Europe has not been fully investigated and donkeys are known to suffer from *Culicoides* bites leading to an allergic dermatitis known as sweet‐itch (Yeruham et al., 1993).

The aim of this study was to conduct light‐suction trap collections around donkeys to determine *Culicoides* species abundance, diversity and feeding behaviour and, therefore, determine whether donkeys may have a potential role in transmission in the event of an AHSV incursion to the United Kingdom.

## METHODS

### Trapping and identification of *Culicoides*

**FIGURE 1:**
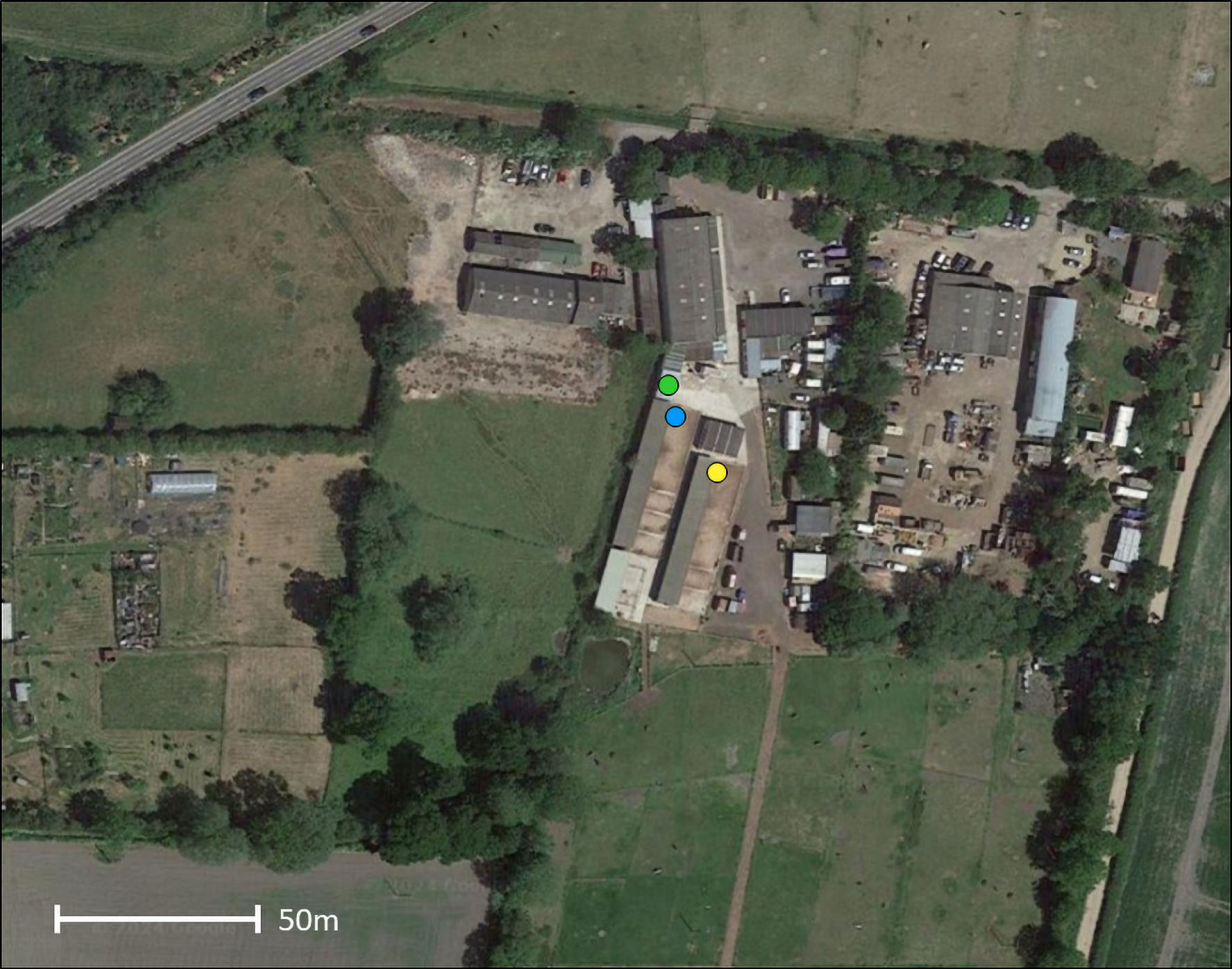
Location of traps at the donkey sanctuary in Oxfordshire, UK, indicated by blue circle: trap 1, yellow circle: trap 2 and green circle: trap 3.

The study site was a donkey sanctuary located in Oxfordshire, United Kingdom (51°36′48.751″ N, 1°10′31.328″ W), situated in a rural area surrounded by countryside at an altitude of 49 m above sea level. At the time of this study, the sanctuary housed around 120 donkeys, a pot‐bellied pig (*Sus domesticus*), chickens (*Gallus gallus domesticus*), goats (*Capra hircus*), dogs (*Canus lupus familiaris*) and a single sheep (*Ovis aries*). Humans were also present on the site as both workers and visitors. Three mains‐powered 8 W ultraviolet (UV) Onderstepoort Veterinary Institute (OVI) light‐suction traps were set up at the sanctuary (Figure 1); two were located outside, attached underneath the roof of open shelters where donkeys were kept at night and during poor weather during the day (traps 1 and 2), and the third trap was set up inside a small barn with an open stable door that housed three donkeys (trap 3). The traps were positioned such that they were not in line of sight of each other and therefore would not interact with each other. Traps were operated overnight from 5 pm until 9 am for a single night each week from 16 April to 18 July 2019. Trapping nights were at approximately weekly intervals, optimising for highest catch based on weather. Arthropods were initially collected in water with a drop of detergent to break the surface tension and then transferred into 70% ethanol the following morning for identification and storage.

Individual *Culicoides* were morphologically identified to species level using existing keys (Campbell & Pelham‐Clinton, 1960; Mathieu et al., 2012) under a light dissecting microscope. Females within the *C. obsoletus/scoticus* complex are morphologically challenging to separate and may constitute several cryptic species. These were grouped together and are hereafter referred to as *C. obsoletus/scoticus* (Mignotte et al., 2020; Pagès & Monteys, 2005). Males of this complex were separated based on the morphology of their genitalia as either *C. obsoletus* or *C. scoticus*, noting these may also constitute several clades (Mignotte et al., 2020). The physiological condition (unpigmented, blood‐fed, gravid or pigmented) of female abdomens of all species was recorded (Dyce, 1969), which provides an indication of age and feeding activity.

### Meteorological data

Historical hourly temperature (°C) and relative humidity (%) data were obtained from the Met Office for the study period. These measurements had been captured by a weather station located at a distance of 2 km from the donkey sanctuary.

### Blood meal analysis

Within a structured sampling approach, a representative subset of 82 blood‐fed female *Culicoides* were selected to include females from each trap and collection date. Blood‐fed females were transferred from 70% ethanol into individual 1.5‐mL Eppendorf tubes containing 200‐μL phosphate buffered saline (PBS) and homogenised with a pestle for 30 s. Following the addition of 180‐μL buffer ATL and 20‐μL proteinase K, each sample was briefly vortexed and incubated overnight at 56°C. DNA was then extracted from the samples using the Qiagen DNeasy Blood and Tissue Kit (Qiagen, UK), following the manufacturer's instructions. The final elution step was performed using 50 μL buffer AE, followed by a further 50 μL buffer AE yielding a final volume of 100 μL of extracted DNA. Extractions were stored at −20°C until further analysis.

A 685 bp region of the cytochrome *c* oxidase subunit I (COI) gene was targeted for blood meal identification using a combination of three forward and three reverse primers (Ivanova et al., 2007). The polymerase chain reaction (PCR) was performed using 5 μL of DNA in a total volume of 25 μL comprising 2.5 μL of PCR buffer, 0.75 μL of 1.5 mM MgCl_2_, 0.5 μL of 200 μM dNTP, 0.25 μL of 0.2 mg/mL bovine serum albumin, 0.1 μL of 1 U Platinum™ *Taq* DNA polymerase (Invitrogen, UK) as well as 0.25 μL of 0.1 μM VF1_t1, VF1d_t1, VR1_t1 and VR1d_t1 primers, 0.5 μL of 0.2 μM VF1i_t1 and VR1i_t1 primers, and 13.9 μL of nuclease‐free water. The PCR cycling conditions were an initial denaturation of 94°C for 2 min, followed by 40 cycles of (1) 94°C for 30 s, (2) 54°C for 30 s, and (3) 72°C for 1 min. A final elongation step of 72°C for 10 min was used. All PCR products were visualised on a 2% agarose gel and samples of the correct size were purified using the MinElute PCR Purification Kit (Qiagen, UK) following manufacturer's instructions. Purified PCR products were sequenced bi‐directionally using M13 primers, by Source Biosciences (Cambridge, UK). Blood meal sequences were compared against all available sequences in GenBank using BLAST and assigned to a host vertebrate species with an identity match of >98% of DNA bases (Brugman et al., 2017).

### Statistical analysis

Statistical analysis of trap catches was conducted using a negative binomial generalised linear model with a log link function. Total *Culicoides* caught per collection was the response variable, while trap, temperature (as a quadratic function) and relative humidity (as a quadratic function) were the explanatory variables. Mean daily temperature (°C) and mean daily relative humidity (%) values were calculated for the day (24‐h period, 00.00–3.59) that the trap was set. Analyses were conducted in the statistical package, R version 4.4.0 (R Core Team, 2024).

## RESULTS

**FIGURE 2:**
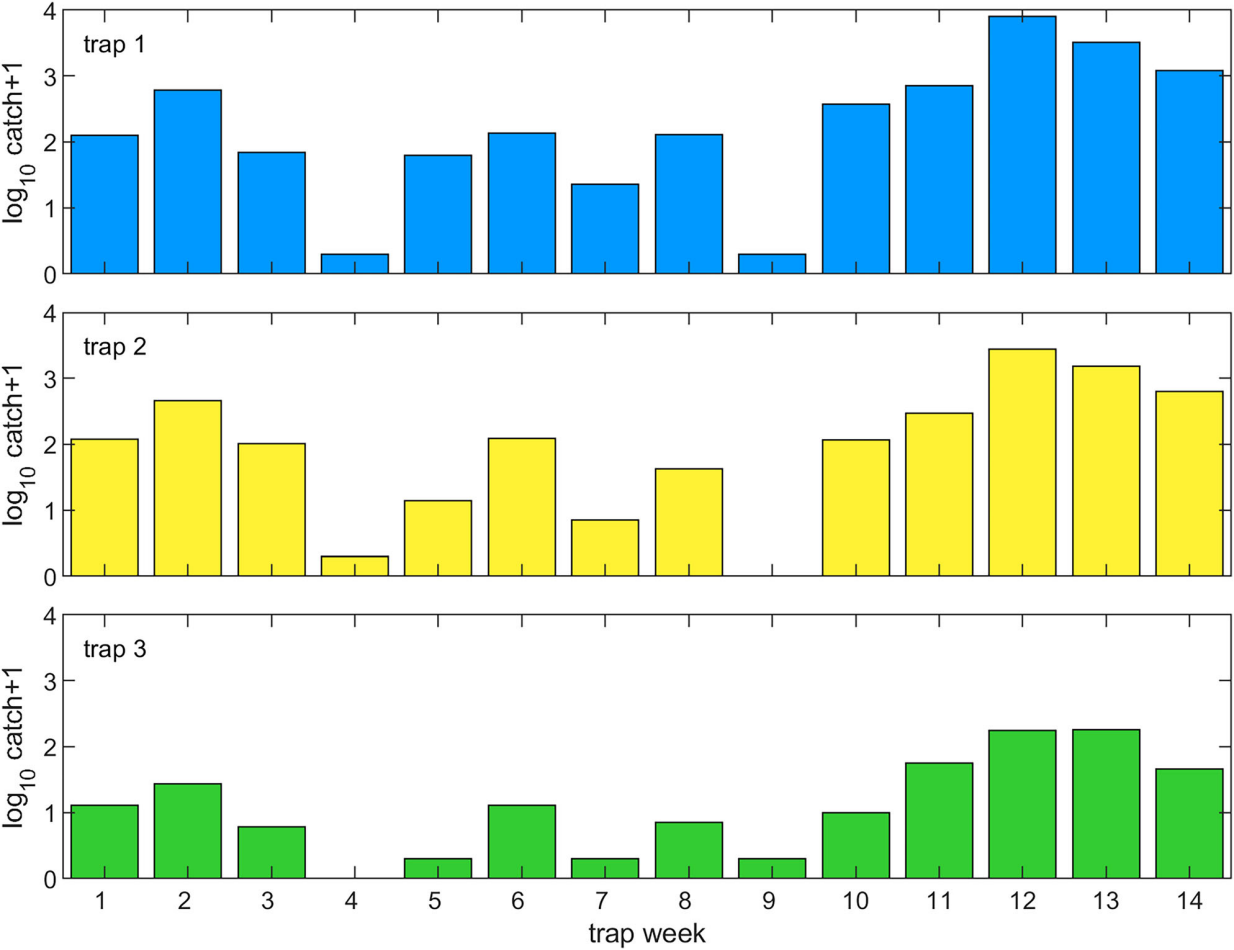
Total *Culicoides* (log_10_ catch+1) caught in light‐suction traps over a 14‐week period at a donkey sanctuary in Oxfordshire, UK.

A total of 21,350 *Culicoides* were collected and identified at the donkey sanctuary over a 14‐week period from a total of 42 trap collections (Table 1, Figure 2). Twenty species of *Culicoides* were collected, with the majority of individuals caught belonging to the *Avaritia* subgenus (98.5%, *n* = 21,028) (*C. obsoletus*, *C. scoticus*, *Culicoides dewulfi* Goetghebuer and *Culicoides chiopterus* Meigen). *Culicoides obsoletus/scoticus* constituted 91.7% of the total *Culicoides* collection (*n* = 19,574). Male *Culicoides* collected accounted for less than 1% of the total collection (*n* = 139). Blood‐fed females made up 1.2% (*n* = 253) of collected females, whereas unpigmented females made up the majority of collected female *Culicoides* (*n* = 11,571, 54.6%) followed by pigmented females (*n* = 7577, 35.7%) and gravid females (*n* = 1810, 8.5%).

**TABLE 1:** Summary of the Culicoides species identified from a 14‐week collection period at a donkey sanctuary in Oxfordshire, UK.

*Culicoides* species	Female	Male	Total
	*n*	*n*	*n* (%)
*C. obsoletus/scoticus*	19,472	102	19,574 (91.7)
*C. chiopterus*	1222	4	1226 (5.4)
*C. pulicaris*	222	6	228 (1.07)
*C. dewulfi*	62	2	64 (0.30)
*C. punctatus*	30	0	30 (0.14)
Sub‐total (putative vector species)	**21,008**	**114**	**21,122 (98.9)**
*C. poperinghensis*	99	5	104 (0.49)
*C. vexans*	42	1	43 (0.20)
*C. nubeculosus*	35	2	37 (0.17)
*C. pictipennis*	10	7	17 (<0.1)
*C. clastrieri*	3	3	6 (<0.1)
*C. puncticollis*	3	2	5 (<0.1)
*C. achrayi*	2	4	6 (<0.1)
*C. festivipennis*	2	1	3 (<0.1)
*C. fagineus*	2	0	2 (<0.1)
*C. impunctatus*	1	0	1 (<0.1)
*C. furcillatus*	1	0	1 (<0.1)
*C. salinarius*	1	0	1 (<0.1)
*C. alazanicus*	1	0	1 (<0.1)
*C. picturatus*	1	0	1 (<0.1)
Sub‐total (other *Culicoides*)	**203**	**25**	**228 (1.07)**
*Total*	**21,211**	**139**	**21,350 (100)**

*Note*: Subtotals and totals given in bold.

A subsample of 82 blood engorged female *Culicoides* were selected for blood meal analysis and of these, 57% (*n* = 47) were successfully matched to a vertebrate host species (Table 2); the remaining 43% failed to amplify. Most host blood meals identified were from donkeys (*n* = 43, 91%). Blood meals from cattle (*n* = 2) and sheep (*n* = 2) were also identified, both from *C. obsoletus/scoticus* females.

**TABLE 2:** Blood meal analysis results of a subsample of blood‐fed female *Culicoides* collected during a 14‐week period (April–July) at a donkey sanctuary in Oxfordshire, UK.

*Culicoides* species	Host species			
	*Equus asinus* (donkey)	*Bos taurus* (cattle)	*Ovis aries* (sheep)	Total
*C. obsoletus/scoticus*	37	2	2	41
*C. dewulfi*	4	‐	‐	4
*C. chiopterus*	2	‐	‐	2
Total	43	2	2	47

The two traps placed outside (traps 1 and 2) collected significantly more *Culicoides* than the trap placed within the stable (trap 3) (*p* < 0.001; Table S1). There was no significant difference between the numbers of *Culicoides* caught in the two outside traps (*p* = 0.38; Table S1). A greater number of species was collected in the two outside traps (16 and 18 species) compared to the inside trap (seven species). The number of *Culicoides* trapped increased with temperature to a maximum at around 21°C after which it declined (Figure S1; Table S1). Similarly, the number of *Culicoides* collected increased with relative humidity to a maximum at around 67%, after which it declined (Figure S1; Table S1).

## DISCUSSION

This is the first study to examine *Culicoides* populations associated with donkeys in the United Kingdom. Previous collections carried out by light trapping and sweep netting at a donkey sanctuary in western Spain that held donkeys, horses and ponies collected six species of *Culicoides*, including *Culicoides fagineus* Edwards and *Culicoides alazanicus* Dzhafarov (González et al., 2022), which were also collected in this UK study. The Spanish study also collected *C. imicola*, which is the principal vector of AHSV in endemic regions, but did not collect *C. obsoletus/scoticus*. The current study has demonstrated that putative *Culicoides* vectors of AHSV within the subgenus *Avaritia* in the United Kingdom are present and abundant around a donkey holding. In addition, a high diversity of other species with unknown vector status was also observed to be present at the study site.

Donkeys have previously been identified as hosts through blood meal analysis carried out in France for *C. obsoletus, Culicoides lupicaris* Downes and Kettle, *Culicoides newsteadi* Austin, *Culicoides punctatus* Meigen and *Culicoides achrayi* Kettle and Lawson (Hadj‐Henni et al., 2015). In the current study, 91% of selected blood‐fed *Culicoides* had fed on donkeys, supporting previous evidence of their ability to feed opportunistically on a wide range of hosts (England et al., 2020). Indeed, it is assumed that the donkey sanctuary provided suitable larval habitats (Harrup et al., 2013) to support the observed large population of *Culicoides*.

Within endemic regions, high incidences of seroprevalence to AHSV have been observed in donkeys. For example, 107 out of 112 (95.5%) domestic donkeys tested by enzyme‐linked immunosorbent assay (ELISA) in The Gambia in 1990 were seropositive to AHSV (Rawlings et al., 1998). In Zimbabwe, seroprevalence in sentinel donkeys was found to be 75% (112 out of 155) (Gordon et al., 2017). Following experimental infection with AHSV‐4, European donkeys have been observed to have viraemia for at least 12 days, but at levels lower than that of ponies (Hamblin et al., 1998), and virus isolation was demonstrated to be possible from AHSV‐infected Moroccan donkeys for up to 17 days post infection (Fassi‐Fihri et al., 1998). It is not known what the minimum level of viraemia in the mammal host must be to ensure systematic infections in susceptible vectors. A greater abundance of vectors may be required to initiate and sustain outbreaks if viraemia in the mammalian host is short‐lived, compared to more susceptible mammalian species.

Here, we have confirmed that a UK donkey holding is able to sustain a high abundance of putative vector species and that these vectors will readily feed on donkeys. Ambient temperatures must also be suitable for the completion of the extrinsic incubation period (EIP) within the vector. Previous studies of AHSV‐4 in *Culicoides sonorensis* Wirth and Jones have demonstrated successful replication and completion of the EIP after 18 days at a constant temperature of 15°C (Wittmann et al., 2002). Indeed, the threshold temperature for AHSV replication within *C. sonorensis* was estimated as 12.6°C, comparable with the threshold for BTV (Carpenter et al., 2011). Given these temperatures are consistently reached during the spring, summer and autumn in the United Kingdom (the main vector active season), and given the current and past outbreaks of BTV in the United Kingdom, it is unlikely that temperature would prevent AHSV transmission in the United Kingdom for a significant proportion of the spring–summer–autumn season.

In the event of an incursion to the United Kingdom, the donkey population would be almost entirely naïve to AHSV, meaning that infections are highly likely to result in at least mild clinical disease. Previous experimental infection studies and serosurveillance have been largely conducted on African donkeys, including the wild ass *E. africanus*. Response to infection of naïve domestic donkeys, *E. asinus*, is poorly understood and there are likely to be differences in susceptibility between breeds, as has been observed for horses (Ahmed et al., 2018). If an incursion occurred during the vector active season, and within the virus transmission season, onwards spread to horses may be expected to occur.

The numbers and species of *Culicoides* collected during this study are comparable to those observed on both cattle farms and horse stables in the United Kingdom, using various trap designs (Baker et al., 2015; Robin et al., 2014; Sanders et al., 2017; Searle et al., 2014). Previously, it has been shown that *Culicoides* can move up to 3 km upwind across the rural landscape in the United Kingdom (Sanders et al., 2017). In this study we were able to infer dispersal of *Culicoides* as two blood‐fed *C. obsoletus/scoticus* were found to have fed on cattle and the nearest cattle farm to the donkey sanctuary was located 3.4 km away. This dispersal distance is based on the straight‐line distance between farm buildings, and it is possible that cattle were grazing nearer to the donkey sanctuary, although the surrounding land is arable, with no suitable grazing or observed presence of cattle. However, this demonstrates mixing of *Culicoides* populations between livestock holdings, which would facilitate AHSV spread.

The number and distribution of donkeys within the United Kingdom is poorly understood. The majority of data on donkeys comes from The Donkey Sanctuary, with most of its donkeys (around 3000) residing in the south west of England, and around 1000 residing in the north west (Cox et al., 2010). A survey of donkey owners estimated that around 2% of donkeys are kept as companion animals alongside horses and ponies (Cox et al., 2010), but this figure is likely to be higher and, where this is the case, the close proximity of equids would facilitate AHSV transmission. As with horses, donkeys are commonly kept at premises other than the registered owner's address, making interpretation of equid distribution data challenging (Lo Iacono et al., 2013).

At the study site, there were a number of additional mammal and bird species that may have contributed to supporting the population of *Culicoides* that we identified. Two blood‐fed *C. obsoletus/scoticus* had fed on sheep, of which a single individual was present at the sanctuary. Only a subset of blood‐fed specimens was processed for blood meal analysis, to enable demonstration of donkey feeding by *Culicoides*, and it is likely that other animals at the site were also being used as a host blood meal. However, the majority (>90%) of the subset that we were able to obtain sequences from had fed on donkeys. The failure to amplify host DNA in almost half of the blood‐fed specimens sampled is not unusual (England et al., 2020) and is likely due to more advanced digestion of the host blood meal (Bellekom et al., 2023). Whilst dogs were present at the site and are known to be a host for *Culicoides* blood‐feeding (Slama et al., 2015; Tomazatos et al., 2020), we did not identify dogs as a host during this study. Clinical presentation of AHS has been observed in domestic dogs in South Africa, without the apparent ingestion of infected meat, suggestive of vector transmission (O'Dell et al., 2018; Oura & El Harrak, 2011).

The use of OVI UV light‐suction traps is a standard method to collect *Culicoides*, and trap comparison studies have shown that it collects more midges than other trap types and is effective at detecting less abundant species (Venter et al., 2009). However, it has also been shown that some species may be under‐represented in light traps (Carpenter et al., 2008). As such, we may have an incomplete picture as to the *Culicoides* present at the site. Additionally, collections were carried out between April and July, precluding the measurement of vector abundance and identification of species that may have been present later in the summer and early autumn.

## CONCLUSIONS

Overall, this study has clearly demonstrated that large populations of *Culicoides*, including putative vectors of AHSV, are present around donkeys in the United Kingdom and are feeding on them. In the event of an incursion of AHSV into the United Kingdom, it is likely that donkeys would be involved in the transmission dynamics, including having the potential to harbour sub‐clinical infection.

## AUTHOR CONTRIBUTIONS

**Zoe Langlands:** Conceptualization; investigation; methodology; writing – original draft; writing – review and editing; formal analysis. **Simon Gubbins:** Methodology; writing – review and editing; writing – original draft; formal analysis; visualization. **Simon Carpenter:** Funding acquisition; writing – review and editing; methodology. **Marion England:** Conceptualization; investigation; writing – original draft; writing – review and editing; methodology; validation; formal analysis; data curation.

## FUNDING INFORMATION

This work was funded by the Defra *Culicoides* Reference Laboratory. ME and SG acknowledge additional funding from UKRI BBSRC (grant code BBS/E/PI/230002C and BBS/E/PI/23NB0003). SC acknowledges additional funding from UKRI BBSRC (grant codes BBS/E/I/00007033, BBS/E/I/00007036 and BBS/E/I/00007039).

## CONFLICT OF INTEREST STATEMENT

The authors declare that they have no conflicts of interest.

## Supporting information


**Figure S1.** Relationship between *Culicoides* abundance and temperature (°C; top panel) or relative humidity (%; bottom panel). Symbols show the observed trap catch, with colour and shape indicating trap location: blue up‐triangle (trap 1); yellow down‐triangle (trap 2); and green circle (trap 3). The solid‐coloured lines show the fitted relationship between abundance and temperature or relative humidity for each trap estimated by a negative binomial generalised linear model.

**Table S1.** Estimated coefficients in a negative binomial generalised linear model for total *Culicoides*. Fulldata.xlsx provides the raw data for *Culicoides* collected during a 14‐week period (April–July) at a donkey sanctuary in Oxfordshire, UK.

## Data Availability

Full data are available as supplementary file, Fulldata.xlsx and have been deposited at VectorByte, https://vectorbyte.crc.nd.edu/vecdyn-detail/952 (Langlands et al., 2026).

## References

[mve70061-bib-0001] Ahmed, A. , Abdalla, M. , Obaid, I. et al. (2018) Epidemiological study of African horse sickness in Sudan. Global Journal of Medical Research: G, 18, 1.

[mve70061-bib-0002] Ayelet, G. , Derso, S. , Jenberie, S. , Tigre, W. , Aklilu, N. , Gelaye, E. et al. (2013) Outbreak investigation and molecular characterization of African horse sickness virus circulating in selected areas of Ethiopia. Acta Tropica, 127(2), 91–96. 23567554 10.1016/j.actatropica.2013.03.018

[mve70061-bib-0003] Baker, T. , Carpenter, S. , Gubbins, S. , Newton, R. , Lo Iacono, G. , Wood, J. et al. (2015) Can insecticide‐treated netting provide protection for equids from *Culicoides* biting midges in the United Kingdom? Parasites & Vectors, 8(1), 604. 26607993 10.1186/s13071-015-1182-x PMC4660720

[mve70061-bib-0004] Barnard, B. (1993) Circulation of African horse sickness virus in zebra (*Equus burchelli*) in the Kruger National Park, South Africa, as measured by the prevalence of antibodies. Onderstepoort Journal of Veterinary Research, 60, 111–117. 8332321

[mve70061-bib-0005] Bellekom, B. , Bailey, A. , England, M. , Langlands, Z. , Lewis, O.T. & Hackett, T.D. (2023) Effects of storage conditions and digestion time on DNA amplification of biting midge (*Culicoides*) blood meals. Parasites & Vectors, 16(1), 13. 36635709 10.1186/s13071-022-05607-x PMC9837887

[mve70061-bib-0006] British Equestrian . (2023) Annual Report. https://www.britishequestrian.org.uk/assets/About%20the%20BEF/Annual%20Report/Annual

[mve70061-bib-0007] Brown, P. (2023) Specieswatch: Donkey work is done as UK population dwindles. https://www.theguardian.com/environment/2023/feb/01/specieswatch-donkey-work-uk-population-dwindles

[mve70061-bib-0008] Brugman, V.A. , Hernández‐Triana, L.M. , England, M.E. , Medlock, J.M. , Mertens, P.P.C. , Logan, J.G. et al. (2017) Blood‐feeding patterns of native mosquitoes and insights into their potential role as pathogen vectors in the Thames estuary region of the United Kingdom. Parasites & Vectors, 10(1), 163. 28347323 10.1186/s13071-017-2098-4 PMC5369192

[mve70061-bib-0009] Bunpapong, N. , Charoenkul, K. , Nasamran, C. , Chamsai, E. , Udom, K. , Boonyapisitsopa, S. et al. (2021) African horse sickness virus serotype 1 on horse farm, Thailand, 2020. Emerging Infectious Diseases, 27(8), 2208–2211. 34287126 10.3201/eid2708.210004 PMC8314833

[mve70061-bib-0010] Campbell, J.A. & Pelham‐Clinton, E.C. (1960) A taxonomic review of the British species of *Culicoides* Latreille (Diptera, Ceratopogonidae). Proceedings of the Royal Society of Edinburgh Section B: Biology, 67(3), 181–302.

[mve70061-bib-0011] Carpenter, S. , Mellor, P.S. , Fall, A.G. , Garros, C. & Venter, G.J. (2017) African horse sickness virus: history, transmission, and current status. Annual Review of Entomology, 62, 343–358. 10.1146/annurev-ento-031616-035010 28141961

[mve70061-bib-0012] Carpenter, S. , Szmaragd, C. , Barber, J. , Labuschagne, K. , Gubbins, S. & Mellor, P. (2008) An assessment of *Culicoides* surveillance techniques in northern Europe: have we underestimated a potential bluetongue virus vector? Journal of Applied Ecology, 45(4), 1237–1245.

[mve70061-bib-0013] Carpenter, S. , Wilson, A. , Barber, J. , Veronesi, E. , Mellor, P. , Venter, G. et al. (2011) Temperature dependence of the extrinsic incubation period of Orbiviruses in *Culicoides* biting midges. PLoS One, 6(11), e27987. 22125649 10.1371/journal.pone.0027987 PMC3220716

[mve70061-bib-0014] Castillo‐Olivares, J. (2021) African horse sickness in Thailand: challenges of controlling an outbreak by vaccination. Equine Veterinary Journal, 53(1), 9–14. 10.1111/evj.13353 PMC7821295 33007121

[mve70061-bib-0015] Cox, R. , Burden, F. , Proudman, C. et al. (2010) Demographics, management and health of donkeys in the UK. Veterinary Record, 166, 552–556. 20435979 10.1136/vr.b4800

[mve70061-bib-0016] Dennis, S.J. , Meyers, A.E. , Hitzeroth, I.I. & Rybicki, E.P. (2019) African horse sickness: a review of current understanding and vaccine development. Viruses, 11, 9. 10.3390/v11090844 PMC6783979 31514299

[mve70061-bib-0017] Dyce, A.L. (1969) The recognition of nulliparous and parous *Culicoides* (Diptera: Ceratopogindae) without dissection. Australian Journal of Entomology, 8(1), 11–15.

[mve70061-bib-0018] England, M.E. , Pearce‐Kelly, P. , Brugman, V.A. , King, S. , Gubbins, S. , Sach, F. et al. (2020) *Culicoides* species composition and molecular identification of host blood meals at two zoos in the UK. Parasites & Vectors, 13(1), 139. 32178710 10.1186/s13071-020-04018-0 PMC7076997

[mve70061-bib-0019] Fassi‐Fihri, O. , El Harrak, M. & Fassi‐Fehri, M.M. (1998) Clinical, virological and immune responses of normal and immunosuppressed donkeys (*Equus asinus africanus*) after inoculation with African horse sickness virus. In: Mellor, P.S. , Baylis, M. , Hamblin, C. , Mertens, P.P.C. & Calisher, C.H. (Eds.) Proceedings of African horse sickness. Vienna: Springer Vienna, pp. 49–56. 10.1007/978-3-7091-6823-3_6 9785495

[mve70061-bib-0020] González, M.A. , Bravo‐Barriga, D. , Fernández, E.B. , Frontera, E. & Ruiz‐Arrondo, I. (2022) Severe skin lesions caused by persistent bites of the stable fly *Stomoxys calcitrans* (Diptera: Muscidae) in a donkey sanctuary of western Spain. Journal of Equine Veterinary Science, 116, 104056. 35753635 10.1016/j.jevs.2022.104056

[mve70061-bib-0021] Gordon, S.J.G. , Bolwell, C. , Rogers, C.W. , Musuka, G. , Kelly, P. , Guthrie, A. et al. (2017) The sero‐prevalence and sero‐incidence of African horse sickness and equine encephalosis in selected horse and donkey populations in Zimbabwe. Onderstepoort Journal of Veterinary Research, 84, 1. 10.4102/ojvr.v84i1.1445 PMC6238669 28582979

[mve70061-bib-0022] Hadj‐Henni, L. , De Meulemeester, T. , Depaquit, J. et al. (2015) Comparison of vertebrate cytochrome b and prepronociceptin for blood meal analyses in *Culicoides* . Frontiers in Veterinary Science, 2, 15. 26664944 10.3389/fvets.2015.00015 PMC4672183

[mve70061-bib-0023] Hamblin, C. , Salt, J.S. , Mellor, P.S. , Graham, S.D. , Smith, P.R. & Wohlsein, P. (1998) Donkeys as reservoirs of African horse sickness virus. In: Mellor, P.S. , Baylis, M. , Hamblin, C. , Mertens, P.P.C. & Calisher, C.H. (Eds.) African horse sickness. Vienna: Springer. 10.1007/978-3-7091-6823-3_5 9785494

[mve70061-bib-0024] Harrup, L.E. , Purse, B.V. , Golding, N. et al. (2013) Larval development and emergence sites of farm‐associated *Culicoides* in the United Kingdom. Medical and Veterinary Entomology, 27(4), 441–449. 23458570 10.1111/mve.12006

[mve70061-bib-0025] Howell, P.G. (1962) The isolation and identification of further antigenic types of African horse sickness virus. Onderstepoort Journal of Veterinary Research, 29, 2.

[mve70061-bib-0026] Ivanova, N.V. , Zemlak, T.S. , Hanner, R.H. et al. (2007) Universal primer cocktails for fish DNA barcoding. Molecular Ecology Notes, 7(4), 544–548.

[mve70061-bib-0027] King, S. , Rajko‐Nenow, P. , Ashby, M. , Frost, L. , Carpenter, S. & Batten, C. (2020) Outbreak of African horse sickness in Thailand, 2020. Transboundary and Emerging Diseases, 67(5), 1764–1767. 32593205 10.1111/tbed.13701

[mve70061-bib-0028] Dataset Langlands, Z. , Gubbins, S. , Carpenter, S. & England, M. (2026) *Culicoides* collected around donkeys in the UK; VecDyn Dataset: 952. 10.1111/mve.70061 PMC13436529 41863060

[mve70061-bib-0029] Lo Iacono, G. , Robin, C.A. , Newton, J.R. , Gubbins, S. & Wood, J.L.N. (2013) Where are the horses? With the sheep or cows? Uncertain host location, vector‐feeding preferences and the risk of African horse sickness transmission in Great Britain. Journal of the Royal Society Interface, 10, 83. 10.1098/rsif.2013.0194 PMC3645429 23594817

[mve70061-bib-0030] Mathieu, B. , Cêtre‐Sossah, C. , Garros, C. , Chavernac, D. , Balenghien, T. , Carpenter, S. et al. (2012) Development and validation of IIKC: an interactive identification key for *Culicoides* (Diptera: Ceratopogonidae) females from the Western Palaearctic region. Parasites & Vectors, 5(1), 137. 22776566 10.1186/1756-3305-5-137 PMC3483010

[mve70061-bib-0031] Maurer, L.M. , Paslaru, A. , Torgerson, P.R. et al. (2021) Vector competence of *Culicoides* biting midges from Switzerland for African horse sickness virus and epizootic haemorrhagic disease virus. Schweizer Archiv für Tierheilkunde, 164(1), 66–70. 10.17236/sat00337 34983740

[mve70061-bib-0032] Meiswinkel, R. , Baylis, M. & Labuschagne, K. (2000) Stabling and the protection of horses from *Culicoides bolitinos* (Diptera: Ceratopogonidae), a recently identified vector of African horse sickness. Bulletin of Entomological Research, 90, 509–515. 11107252 10.1017/s0007485300000626

[mve70061-bib-0033] Mellor, P.S. , Boned, J. , Hamblin, C. & Graham, S. (1990) Isolations of African horse sickness virus from vector insects made during the 1988 epizootic in Spain. Epidemiology & Infection, 105(2), 447–454. 2209746 10.1017/s0950268800048020 PMC2271884

[mve70061-bib-0034] Mellor, P.S. & Boorman, J. (1995) The transmission and geographical spread of African horse sickness and bluetongue viruses. Annals of Tropical Medicine & Parasitology, 89(1), 1–15. 7741589 10.1080/00034983.1995.11812923

[mve70061-bib-0035] Mellor, P.S. & Hamblin, C. (2004) African horse sickness. Veterinary Research, 35(4), 445–466. 15236676 10.1051/vetres:2004021

[mve70061-bib-0036] Mignotte, A. , Garros, C. , Gardès, L. , Balenghien, T. , Duhayon, M. , Rakotoarivony, I. et al. (2020) The tree that hides the forest: cryptic diversity and phylogenetic relationships in the Palaearctic vector *Obsoletus/Scoticus* complex (Diptera: Ceratopogonidae) at the European level. Parasites & Vectors, 13(1), 265. 32434592 10.1186/s13071-020-04114-1 PMC7238629

[mve70061-bib-0037] O'Dell, N. , Arnot, L. , Janisch, C.E. & Steyl, J.C.A. (2018) Clinical presentation and pathology of suspected vector transmitted African horse sickness in south African domestic dogs from 2006 to 2017. Veterinary Record, 182(25), 715. 29519857 10.1136/vr.104611

[mve70061-bib-0038] Oura, C.A.L. & El Harrak, M. (2011) Midge‐transmitted bluetongue in domestic dogs. Epidemiology and Infection, 139(9), 1396–1400. 21044402 10.1017/S0950268810002396

[mve70061-bib-0039] Pagès, N. & Monteys, V.S.i. (2005) Differentiation of *Culicoides obsoletus* and *Culicoides scoticus* (Diptera: Ceratopogonidae) based on mitochondrial cytochrome oxidase subunit I. Journal of Medical Entomology, 42(6), 1026–1034. 16465744 10.1093/jmedent/42.6.1026

[mve70061-bib-0040] Purse, B.V. , Carpenter, S. , Venter, G.J. , Bellis, G. & Mullens, B.A. (2015) Bionomics of temperate and tropical *Culicoides* midges: knowledge gaps and consequences for transmission of *Culicoides*‐borne viruses. Annual Review of Entomology, 60, 373–392. 10.1146/annurev-ento-010814-020614 25386725

[mve70061-bib-0041] R Core Team . (2024) R: a language and environment for statistical computing. Vienna, Austria: R Foundation for Statistical Computing.

[mve70061-bib-0042] Rawlings, P. , Snow, W.F. , Boorman, J. et al. (1998) *Culicoides* in relation to transmission of African horse sickness virus in The Gambia. Medical and Veterinary Entomology, 12(2), 155–159. 9622369 10.1046/j.1365-2915.1998.00094.x

[mve70061-bib-0043] Robin, M. , Archer, D. , Garros, C. , Gardès, L. & Baylis, M. (2014) The threat of midge‐borne equine disease: investigation of *Culicoides* species on UK equine premises. Veterinary Record, 174(12), 301. 24508765 10.1136/vr.102151

[mve70061-bib-0044] Sanders, C.J. , Harrup, L.E. , Tugwell, L.A. , Brugman, V.A. , England, M. & Carpenter, S. (2017) Quantification of within‐ and between‐farm dispersal of *Culicoides* biting midges using an immunomarking technique. Journal of Applied Ecology, 54(5), 1429–1439. 29104309 10.1111/1365-2664.12875 PMC5655569

[mve70061-bib-0045] Scacchia, M. , Molini, U. , Marruchella, G. , Maseke, A. , Bortone, G. , Cosseddu, G.M. et al. (2015) African horse sickness outbreaks in Namibia from 2006 to 2013: clinical, pathological and molecular findings. Veterinaria Italiana, 51(2), 123–130. 26129663 10.12834/VetIt.200.617.3

[mve70061-bib-0046] Searle, K.R. , Barber, J. , Stubbins, F. , Labuschagne, K. , Carpenter, S. , Butler, A. et al. (2014) Environmental drivers of *Culicoides* phenology: how important is species‐specific variation when determining disease policy? PLoS One, 9(11), e111876. 25386940 10.1371/journal.pone.0111876 PMC4227682

[mve70061-bib-0047] Slama, D. , Haouas, N. , Mezhoud, H. , Babba, H. & Chaker, E. (2015) Blood meal analysis of *Culicoides* (Diptera: Ceratopogonidae) in Central Tunisia. PLoS One, 10(3), e0120528. 25793285 10.1371/journal.pone.0120528 PMC4368833

[mve70061-bib-0048] Theiler, A. (1921) African horse sickness (pestis equorum). Science Bulletin, 19, 1–29.

[mve70061-bib-0049] Tomazatos, A. , Jöst, H. , Schulze, J. , Spînu, M. , Schmidt‐Chanasit, J. , Cadar, D. et al. (2020) Blood‐meal analysis of *Culicoides* (Diptera: Ceratopogonidae) reveals a broad host range and new species records for Romania. Parasites & Vectors, 13(1), 79. 32066493 10.1186/s13071-020-3938-1 PMC7027113

[mve70061-bib-0050] Venter, G.J. , Labuschagne, K. , Hermanides, K.G. , Boikanyo, S.N.B. , Majatladi, D.M. & Morey, L. (2009) Comparison of the efficiency of five suction light traps under field conditions in South Africa for the collection of *Culicoides* species. Veterinary Parasitology, 166(3), 299–307. 19758757 10.1016/j.vetpar.2009.08.020

[mve70061-bib-0051] Wittmann, E. , Mellor, P. & Baylis, M. (2002) Effect of temperature on the transmission of orbiviruses by the biting midge. Culicoides Sonorensis. Medical and Veterinary Entomology, 16(2), 147–156. 12109708 10.1046/j.1365-2915.2002.00357.x

[mve70061-bib-0052] WOAH . (2020a) African horse sickness in Malaysia (update and control measures).

[mve70061-bib-0053] WOAH . (2020b) Technical Disease Card: African Horse Sickness.

[mve70061-bib-0054] Yeruham, I. , Braverman, Y. & Orgad, U. (1993) Field observations in Israel on hypersensitivity in cattle, sheep and donkeys caused by *Culicoides* . Australian Veterinary Journal, 70(9), 348–352. 8240175 10.1111/j.1751-0813.1993.tb00883.x

