# Supplementary material for: Culicoides biting midges (Diptera: Ceratopogonidae) feeding on donkeys in the United Kingdom, with reference to the risk of transmission and persistence of African horse sickness virus
Source: Med Vet Entomol. 2026 Mar 20;40(3):560–7. doi: 10.1111/mve.70061 (PMC13436529; doi:10.1111/mve.70061)
Supplement: Supplementary file 1 [file MVE-40-560-s002.docx]

**Table S1.** Estimated coefficients in a negative binomial generalised linear model for total *Culicoides*.

|  | estimate | 95% confidence interval |
| --- | --- | --- |
| intercept | -18.32 | (-38.21, 4.06) |
| *trap* |  |  |
| 1 | 0 (baseline) | - |
| 2 | -0.60 | (-1.52, 0.32) |
| 3 | -2.89 | (-3.84, -1.94) |
| *temperature* |  |  |
| linear (*T*) | 1.11 | (0.14, 1.99) |
| quadratic (*T*^2^) | -0.03 | (-0.06, 0.01) |
| *humidity* |  |  |
| linear (*H*) | 0.45 | (-0.10, 0.90) |
| quadratic (*H*^2^) | -0.003 | (-0.006, 0.0002) |
| dispersion parameter | 0.69 | (0.42, 0.97) |
